# Postpartum Psychiatric Outcomes and Sick Leave After Discontinuing SSRI or SNRI in Pregnancy

**DOI:** 10.1001/jamanetworkopen.2024.38269

**Published:** 2024-10-08

**Authors:** Carolyn E. Cesta, Johan Reutfors, Jacqueline M. Cohen, Julia Eriksson, Kari Furu, Helga Zoega, Laura Pazzagli

**Affiliations:** 1 Centre for Pharmacoepidemiology, Department of Medicine Solna, Karolinska Institutet, Stockholm, Sweden; 2 Department of Chronic Diseases, Norwegian Institute of Public Health, Oslo, Norway; 3 Centre for Fertility and Health, Norwegian Institute of Public Health, Oslo, Norway; 4 Centre of Public Health Sciences, Faculty of Medicine, University of Iceland, Reykjavik, Iceland; 5 School of Population Health, Faculty of Medicine and Health, University of New South Wales Sydney, Sydney, Australia; 6 Clinical Epidemiology Division, Department of Medicine Solna, Karolinska Institutet, Stockholm, Sweden

## Abstract

**Importance:**

Selective serotonin reuptake inhibitors (SSRIs) and serotonin-norepinephrine reuptake inhibitors (SNRIs) are consistently reported to be discontinued by approximately half of pregnant women. Little is known about how this may be associated with postpartum psychiatric health.

**Objective:**

To investigate associations of SSRI or SNRI discontinuation in pregnant women with depression or anxiety and psychiatric health and sick leave absence after childbirth.

**Design, Setting, and Participants:**

This population-based cohort study was conducted between 2006 and 2019 using data from Swedish population-based registers. Pregnant women with a filled prescription of an SSRI or SNRI in the 90 days before pregnancy without recorded comorbid or severe psychiatric conditions were included. Analyses were performed in November 2023.

**Exposures:**

K-means for longitudinal data was used to cluster trajectories of SSRI and SNRI use during pregnancy, resulting in 2 trajectory groups based on the number of days covered, defined as continued and discontinued use groups.

**Main Outcomes and Measures:**

The primary outcome was psychiatric-related hospitalizations by 90 days after childbirth. Secondary outcomes included psychiatric-related outpatient visits, self-harm and suicide, and any-cause mortality by 90 days after childbirth and all outcomes plus sick leave absence by 1.5 years after childbirth.

**Results:**

Among 27 773 pregnant women (17 241 aged ≥30 years [62.1%] at childbirth), 13 184 women (47.5%) had discontinued SSRI or SNRI use and 14 589 individuals (52.5%) had continued use. Individuals in the discontinued compared with continued use group were younger (5588 women [42.4%] vs 4944 women [33.9%] aged <30 years), less educated (4281 women [32.5%] vs 5821 women [39.9%] who completed postsecondary education or above), and more likely to have smoked in early pregnancy (1445 individuals [11.0%] vs 1180 individuals [8.1%]), been born in a non-Nordic country (1641 individuals [12.4%] vs 975 individuals [6.7%]), and used anxiolytics (1301 individuals [9.9%] vs 1119 individuals [7.7%]) and hypnotics and sedatives (1609 individuals [12.2%] vs 1510 individuals [10.4%]). Psychiatric-related hospitalizations occurred in 49 individuals (0.4%) in the discontinued vs 59 individuals (0.5%) in the continued use group in the 90 days after childbirth, with an adjusted hazard ratio (aHR) of 1.28 (95% CI, 0.85-1.91), while at 1.5 years after childbirth, the aHR was 0.81 (95% CI, 0.66-1.00). Lower hazard rates for psychiatric-related outpatient visits in the discontinued vs continued use group at 90 days (aHR, 0.59; 95% CI, 0.53-0.66) and 1.5 years (aHR, 0.60; 95% CI, 0.57-0.64) after childbirth were found. No difference in sick leave absence was found; however, individuals who discontinued had fewer days of sick leave by 1.5 years after childbirth than those who continued (mean [SD], 44.6 [70.6] days vs 53.1 [82.3] days).

**Conclusions and Relevance:**

In this study, approximately half of pregnant women discontinued SSRIs or SNRIs, and discontinuation during pregnancy was not associated with adverse psychiatric-related outcomes, including hospitalizations, outpatient visits, suicidal behavior, or sick leave absence in the 90 days or 1.5 years after childbirth.

## Key Points

**Question:**

Do women with depression or anxiety without comorbid or severe psychiatric conditions who discontinue selective serotonin reuptake inhibitors (SSRIs) or serotonin-norepinephrine reuptake inhibitors (SNRIs) in pregnancy have a higher prevalence of adverse postpartum psychiatric-related outcomes?

**Findings:**

In this Swedish cohort of 27 773 women with prepregnancy SSRI or SNRI use, 47.5% discontinued use during pregnancy. Compared with continuation, SSRI or SNRI discontinuation was not associated with adverse psychiatric outcomes, including hospitalizations, outpatient visits, suicidal behavior, or sick leave absence by 90 days or 1.5 years after childbirth.

**Meaning:**

This study found that for women without comorbid or severe psychiatric conditions who discontinued SSRI or SNRI use in pregnancy, there was no increase in adverse postpartum psychiatric outcomes.

## Teaser

This cohort study investigates the association of discontinuing selective serotonin reuptake inhibitors (SSRIs) or serotonin-norepinephrine reuptake inhibitors (SNRIs) during pregnancy with postpartum psychiatric outcomes and sick leave absence after childbirth among women in Sweden.

## Introduction

Antidepressant use in reproductive-aged women has increased over the last decades,^1^ but there is little guidance on the management of treatment for women who become pregnant.^2^ Selective serotonin reuptake inhibitors (SSRIs) and serotonin-norepinephrine reuptake inhibitors (SNRIs) are the most commonly used classes of antidepressants in pregnant women,^3^ and it is consistently reported that approximately half of women discontinue use before or during pregnancy.^4,5^ While most pregnant women use SSRIs or SNRIs for depression or anxiety, such medications are also used in women being treated for other psychiatric conditions (eg, insomnia, obsessive-compulsive disorder, eating disorders, and bipolar or psychotic disorders),^3^ which are known to carry a higher risk for adverse postpartum psychiatric outcomes.^6^

Previous studies investigating risks associated with discontinuation of treatment during pregnancy included all individuals using SSRIs or SNRIs before pregnancy regardless of indication.^7,8,9^ However, evidence suggests that the severity of the maternal psychiatric burden rather than the discontinuation of antidepressants per se is associated with the risk of adverse psychiatric events.^10^ To address the potential bias resulting from confounding by indication, this study aimed to investigate the association between SSRI or SNRI discontinuation in pregnancy and psychiatric-related hospitalizations and outpatient visits, suicidal behavior, and sick leave absence in the postpartum period in a cohort of women with depression or anxiety without recorded comorbid or severe psychiatric conditions.

## Methods

This cohort study was approved by the Swedish Ethical Review Authority. Informed consent for inclusion in the national registers is not required. The study follows the Strengthening the Reporting of Observational Studies in Epidemiology (STROBE) reporting guideline for observational studies.

### Data Sources, Study Design, and Population

This population register–based cohort study included data from Swedish national demographic and health registers linked via the unique personal identification number between 2006 and 2019.^11^ In this study, we refer to biologic sex and use the term *pregnant woman* to define pregnant females of any gender identity. Gender identity was not recorded in the databases. Pregnancies were identified from the Medical Birth Register, which contains data on all deliveries in Sweden since 1973.^12^ The National Patient Register contains full coverage of diagnoses made in inpatient care since 1987 and for outpatient hospital-based specialist care since 2001. Since July 1, 2005, the Prescribed Drug Register has collected information on prescriptions dispensed at community pharmacies for the entire Swedish population.^13^ The Cause of Death Register is a complete register on all deaths in Sweden since 1952.^14^ Data on education were obtained from the Swedish Longitudinal Integrated Database for Health Insurance and Labour Market Studies at Statistics Sweden,^15^ and data on sick leave absence and disability pension were obtained from the Swedish Social Insurance Agency database, MiDAS.

The study population included singleton pregnancies resulting in live birth or stillbirth after 22 weeks of gestation July 31, 2006, to June 30, 2017, in women with 1 or more SSRI or SNRI prescription fill in the 90 days before the first day of the last menstrual period (LMP). Pregnancies were required to have available data from 1 year prior to LMP to 1.5 years after childbirth. Furthermore, to restrict the study population to women without severe depression or anxiety and without comorbid psychiatric disorders, pregnancies were excluded if women had any recorded psychiatric diagnosis from specialist care in the year before LMP, except for outpatient diagnoses of mild or moderate unipolar depression and anxiety, or any prescription fill in the 90 days prior to LMP of non–SSRI or SNRI antidepressants, antipsychotics, or antiepileptics (eTable 1 in Supplement 1). Analyses were performed in November 2023.

### Exposure

SSRI or SNRI use during pregnancy was defined by trajectories of number of gestational days covered by prescription fills. Pregnancies were clustered via the unsupervised k-means algorithm to identify women with similar levels of SSRI or SNRI use after LMP. In detail, all SSRI or SNRI prescriptions filled from LMP to birth were used to calculate the duration of use during pregnancy under the assumption that women consumed 1 tablet per day of each drug included. The assumption was based on an internal investigation into the free text information containing the intended dose or number of tablets per day indicated by the prescriber and recorded in the Prescribed Drug Register, which reflected that most individuals taking SSRIs or SNRIs took 1 tablet per day. Each pregnancy was divided into nine 30-day periods from LMP to day 270 of gestation (38.5 weeks). For each period, the number of gestational days covered by medication was calculated. When a pregnancy ended before gestational day 270, the remainder of the days in the current 30-day period and the remaining 30-day periods were set to missing.^16^

Clusters of individual SSRI or SNRI patterns of use during pregnancy (trajectories) were identified with the k-means clustering algorithm for longitudinal data implemented in the R statistical software package kml, with the starting condition assigning k random individuals to k groups and allowing k means to run from 2 to 6 clusters 100 times each.^17,18^ Selection of the number of clusters was based on optimization of 3 quality criterion, Calinski-Harabasz, Ray Turi, and Davies Bouldin,^19^ all of which were in agreement that 2 clusters were the optimal partition.

Pregnancies were clustered in 2 exposure groups. The trajectories group with the highest mean number of days covered during pregnancy was defined as the SSRI or SNRI continued use group, while the trajectories group with the lowest mean number of days covered was defined as the SSRI or SNRI discontinued use group.

### Outcomes

The primary outcome of interest was psychiatric-related hospitalizations in the 90 days after childbirth. Secondary outcomes included psychiatric-related hospitalizations in the 1.5 years after childbirth and outpatient visits, self-harm and suicide, and any cause mortality by 90 days and 1.5 years after childbirth. Additionally, sick leave absence, number of days on sick leave, and indication for sick leave absence in the 1.5 years after childbirth were investigated (eTable 2 in Supplement 1). Women on long-term disability are not eligible for paid sick leave and were excluded from sick leave analyses.

### Statistical Analysis

Pregnancies clustered in the SSRI or SNRI discontinued use group were compared with those in the SSRI or SNRI continued use group, and outcomes were analyzed with a time-to-event analysis (Cox proportional hazards) estimating hazard ratios (HRs) and 95% CIs. The number of days on sick leave was analyzed with a count data regression analysis (Poisson) estimating incidence rate ratios (IRRs) with 95% CIs. Models were adjusted for key confounders identified a priori based on clinical knowledge and findings from previous studies, including year of childbirth, maternal age, parity, country of birth, cohabitation status, early pregnancy smoking, highest achieved maternal education in the year of childbirth, early pregnancy body mass index (calculated as weight in kilograms divided by height in meters squared), sick leave absence in the year before the start of pregnancy (included only for sick leave outcomes), and prepregnancy use of anxiolytics (ATC code N05B), hypnotics and sedatives (ATC codes N05C, R06AD02, and R06AD01), or stimulants (ATC codes N06B and C02AC02) identified by 1 or more filled prescriptions from 90 days before LMP to LMP. In the case of missing data, a missing indicator category was included in the variable definition. Analyses were conducted using R statistical software version 4.3.2 (R Project for Statistical Computing).^20^

## Results

**Figure 1.**
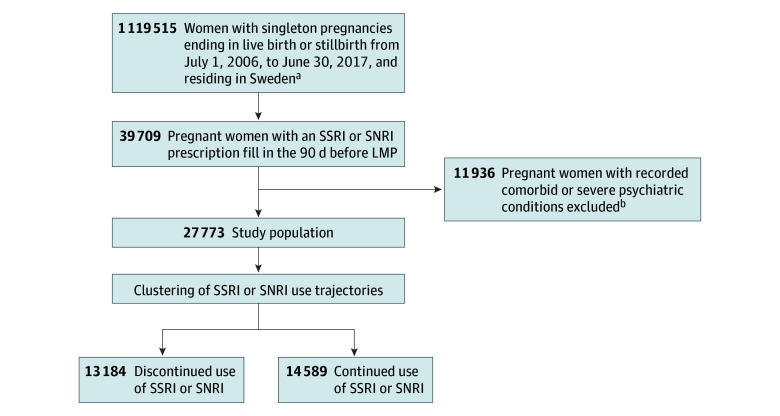
Study Flowchart LMP indicates first day of the last menstrual period; SNRI, serotonin-norepinephrine reuptake inhibitor; SSRI, selective serotonin reuptake inhibitor. ^a^Residing in Sweden from 1 year before LMP to 1.5 years after childbirth. ^b^Defined as any psychiatric diagnosis in the year before LMP (except outpatient diagnoses of mild or moderate unipolar depression and anxiety) or any prescription fill in the 90 days prior to LMP of non–SSRI or SNRI antidepressants, antipsychotics, or antiepileptics.

In total, 39 709 women had an SSRI or SNRI prescription fill in the 90 days prior to LMP. Among them, 11 936 women (30.1%) had a recorded comorbid or severe psychiatric condition and were excluded (Figure 1), resulting in a study cohort of 27 773 pregnant women (17 241 aged ≥30 years [62.1%] at childbirth). The k-means algorithm clustered the study population into 14 589 individuals (52.5%) in the SSRI or SNRI continued use group and 13 184 individuals (47.5%) in the SSRI or SNRI discontinued use group (eFigure in Supplement 1). Individuals who discontinued compared with continued were younger (5588 women [42.4%] vs 4944 women [33.9%] aged <30 years) and less educated (4281 women [32.5%] vs 5821 women [39.9%] who completed postsecondary education or above) and more likely to have smoked in early pregnancy (1445 individuals [11.0%] vs 1180 individuals [8.1%]), been born in a non-Nordic country (1641 individuals [12.4%] vs 975 individuals [6.7%]), and have used anxiolytics (1301 individuals [9.9%] vs 1119 individuals [7.7%]) and hypnotics and sedatives (1609 individuals [12.2%] vs 1510 individuals [10.4%]) (Table 1). In the discontinued use group, 6128 individuals (46.5%) reinitiated SSRIs or SNRIs after childbirth, with a median (IQR) of 162 (70-310) days between childbirth and the next SSRI or SNRI prescription fill.

**Table 1.** Characteristics of Study Population

Characteristic	Pregnant women, No. (%)		
	Total (N = 27 773)	SSRI or SNRI continued (n = 14 589 [52.5%])	SSRI or SNRI discontinued (n = 13 184 [47.5%])
Year of childbirth			
2006-2008	5120 (18.4)	1940 (13.3)	3180 (24.1)
2009-2013	11 866 (42.7)	6306 (43.2)	5560 (42.2)
2014-2017	10 787 (38.8)	6343 (43.5)	4444 (33.7)
Maternal age at childbirth, y			
<20	276 (1.0)	70 (0.5)	206 (1.6)
20-24	2792 (10.1)	1105 (7.6)	1687 (12.8)
25-29	7464 (26.9)	3769 (25.8)	3695 (28.0)
30-34	9575 (34.5)	5276 (36.2)	4299 (32.6)
35-39	6115 (22)	3533 (24.2)	2582 (19.6)
40-44	1494 (5.4)	805 (5.5)	689 (5.2)
≥45	57 (0.2)	31 (0.2)	26 (0.2)
Smoking, early pregnancy			
No	24 084 (86.7)	12 852 (88.1)	11 232 (85.2)
Yes	2625 (9.5)	1180 (8.1)	1445 (11.0)
Missing	1064 (3.8)	557 (3.8)	507 (3.8)
BMI, early pregnancy			
<18.5	515 (1.9)	259 (1.8)	256 (1.9)
18.5-24	13 876 (50.0)	7236 (49.6)	6640 (50.4)
25-29	7090 (25.5)	3764 (25.8)	3326 (25.2)
30-34	2854 (10.3)	1525 (10.5)	1329 (10.1)
≥35	1471 (5.3)	812 (5.6)	659 (5.0)
Missing	1967 (7.1)	993 (6.8)	974 (7.4)
Maternal country of birth			
Nordic	25 157 (90.6)	13 614 (93.3)	11 543 (87.6)
Non-Nordic	2616 (9.4)	975 (6.7)	1641 (12.4)
Parity			
0	12 294 (44.3)	6287 (43.1)	6007 (45.6)
1	9481 (34.1)	5348 (36.7)	4133 (31.3)
2	4236 (15.3)	2090 (14.3)	2146 (16.3)
3	1206 (4.3)	593 (4.1)	613 (4.6)
≥4	556 (2.0)	271 (1.9)	285 (2.2)
Cohabitating	24 059 (86.6)	12 848 (88.1)	11 211 (85.0)
Highest maternal education			
Compulsory	2955 (10.6)	1241 (8.5)	1714 (13.0)
Secondary	14 601 (52.6)	7478 (51.3)	7123 (54.0)
Postsecondary	9928 (35.7)	5728 (39.3)	4200 (31.9)
Postgraduate	174 (0.6)	93 (0.6)	81 (0.6)
Missing	117 (0.4)	49 (0.3)	68 (0.5)
Comedication^a^			
Anxiolytic	2420 (8.7)	1119 (7.7)	1301 (9.9)
Hypnotic or sedative	3119 (11.2)	1510 (10.4)	1609 (12.2)
Stimulant	139 (0.5)	79 (0.5)	60 (0.5)
Substance use disorder	65 (0.2)	36 (0.2)	29 (0.2)
Sick leave absence			
Before pregnancy	8439 (30.4)	4164 (28.5)	4275 (32.4)
During pregnancy	13 229 (47.6)	7040 (48.3)	6189 (46.9)

Abbreviation: BMI, body mass index (calculated as weight in kilograms divided by height in meters squared).

^a^ Defined as a filled prescription from 90 days before the first day of the last menstrual period to the last menstrual period. Anxiolytics include ATC code N05B. Hypnotics and sedatives include ATC codes N05C, R06AD02, and R06AD01. Stimulants include ATC codes N06B and C02AC02.

**Figure 2.**
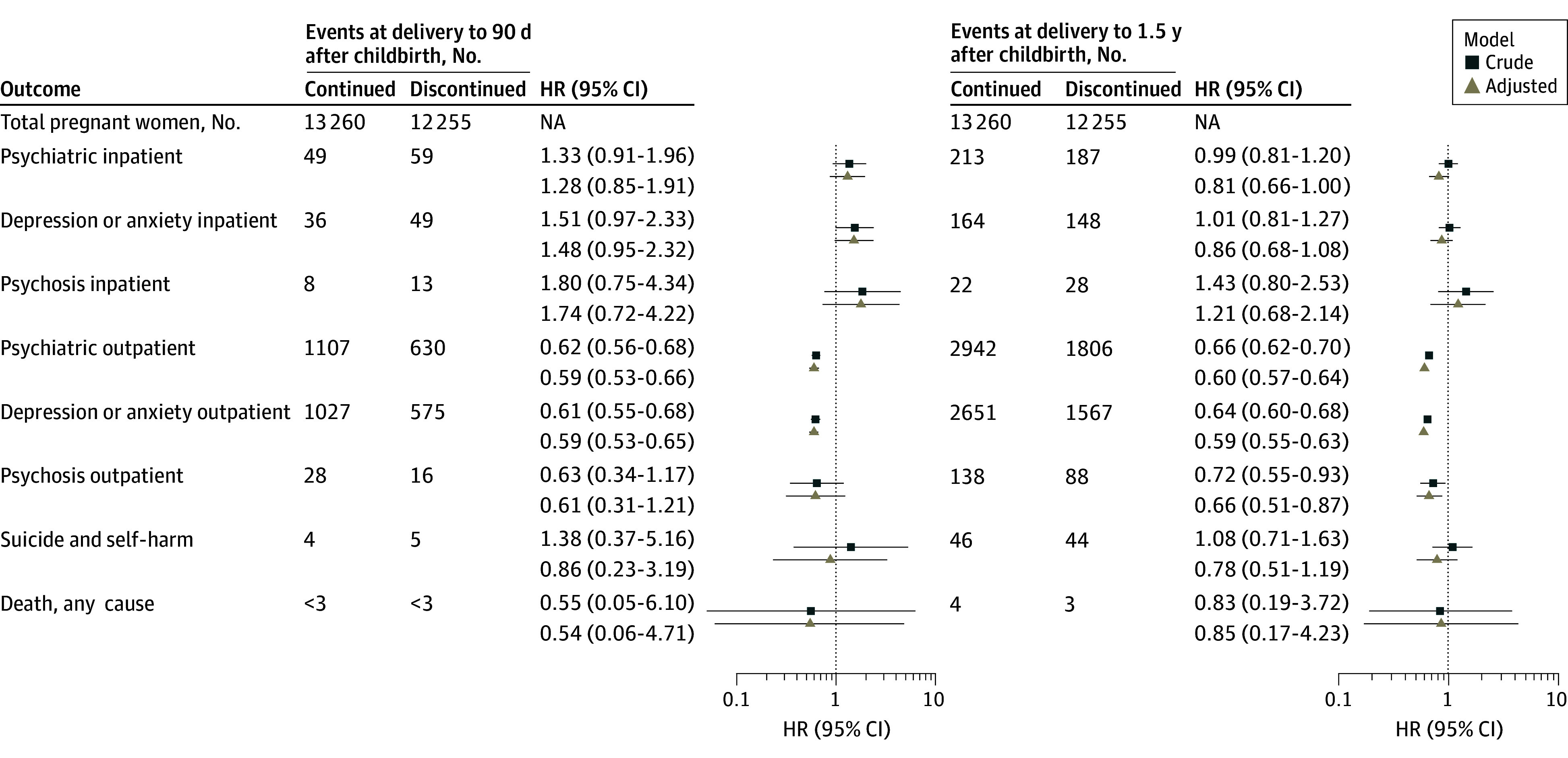
Number of Events and Hazard Ratios of Adverse Psychiatric Outcomes Continued and discontinued use groups refer to selective serotonin reuptake inhibitor or serotonin-norepinephrine reuptake inhibitor use in pregnancy. Models were adjusted for year of childbirth, maternal age, parity, country of birth, cohabitation status, smoking in early pregnancy, comedication (anxiolytics, hypnotics and sedatives, and stimulants), education, and body mass index (calculated as weight in kilograms divided by height in meters squared). Counts less than 3 are not shown for privacy protection. HR indicates hazard ratio; NA, not applicable.

By 90 days after childbirth, psychiatric-related hospitalizations had occurred in 49 individuals (0.4%) in the discontinued vs 59 individuals (0.5%) in the continued use group. The point estimate showed a higher hazard rate for the discontinued vs continued use group at 90 days after childbirth (adjusted HR [aHR], 1.28; 95% CI, 0.85-1.91), but this was attenuated and less than 1 at 1.5 years after childbirth (aHR, 0.81; 95% CI, 0.66-1.00). A lower hazard rate was detected for the discontinued vs continued use group for psychiatric-related outpatient visits at 90 days (aHR, 0.59; 95% CI, 0.53-0.66) and 1.5 years (aHR, 0.60; 95% CI, 0.57-0.64) after childbirth (Figure 2). No association between SSRI or SNRI discontinuation and self-harm or suicide was found (90 days: aHR, 0.86; 95% CI, 0.23-3.19; 1.5 years: aHR, 0.78; 95% CI, 0.51-1.19). Other outcomes also had no association but had high HRs and wide CIs with a small number of events (eg, hospitalization for psychoses at 90 days after childbirth: aHR, 1.74; 95% CI, 0.72-4.22).

No difference between the SSRI or SNRI discontinued vs continued use groups was observed in whether women had a sick leave absence (aHR, 0.93; 95% CI, 0.85-1.01). However, individuals in the discontinued use group had fewer days of sick leave during the 1.5 years after childbirth compared with those in the continued use group (mean [SD], 44.6 [70.6] days vs 53.1 [82.3] days; adjusted IRR, 0.86; 95% CI, 0.76-0.98) (Table 2).

**Table 2.** Sick Leave Absence in the 1.5 Years After Childbirth

Absence measure	Pregnant women, No. (%)		Outcome (95% CI)	
	Continued (n = 13 260)^a^	Discontinued (n = 12 255)^a^	Crude	Adjusted^b^
Any sick leave absence	1273 (9.6)	1055 (8.6)	HR, 0.90 (0.83-0.97)	HR, 0.93 (0.85-1.01)
Length of sick leave absence, mean (SD), d	53.1 (82.3)	44.6 (70.6)	IRR, 0.84 (0.74-0.95)	IRR, 0.86 (0.76-0.98)
Sick leave absence >90 d	238 (18.7)^c^	146 (13.8)^c^	NA	NA
Psychiatric indication for sick leave absence	426 (33.5)^c^	345 (32.7)^c^	NA	NA

Abbreviations: HR, hazard ratio; IRR, incidence rate ratio; NA, not applicable.

^a^ Continued and discontinued use groups refer to selective serotonin reuptake inhibitor or serotonin-norepinephrine reuptake inhibitor use in pregnancy.

^b^ Models were adjusted for year of childbirth, maternal age, parity, country of birth, cohabitation, smoking in early pregnancy, education, body mass index (calculated as weight in kilograms divided by height in meters squared), comedication (anxiolytics, hypnotics and sedatives, and stimulants), and sick leave absence 1 year prior to the last menstrual period (for sick leave use models only).

^c^ Percentages of individuals using sick leave are descriptive only.

## Discussion

This Swedish population-based register cohort study investigated women who discontinued or continued SSRIs or SNRIs in pregnancy. There was no association between discontinued use and adverse psychiatric-related outcomes, including hospitalizations or outpatient visits, suicidal behavior, or sick leave absence during the 90 days and 1.5 years after childbirth, compared with continued SSRI or SNRI use. Compared with all women taking SSRIs or SNRIs, the cohort was restricted to a more homogenous group of women using first-line antidepressants (SSRIs or SNRIs) with a similar level of depression and anxiety at baseline to overcome potential confounding by indication bias, which can result from differences in severity or comorbid psychiatric conditions between groups. Notably, 69.9% of individuals taking SSRIs or SNRIs before pregnancy were included in the study cohort, highlighting that this is an important patient group for which evidence on risks and benefits of SSRI or SNRI use or discontinuation in pregnancy is needed.

Previous studies on discontinuation in pregnancy included all individuals taking antidepressants before pregnancy regardless of indication and have reported inconsistent results for adverse psychiatric events after childbirth. Pregnant women who discontinued antidepressants early and later in pregnancy have previously been reported to have a lower probability of psychiatric emergencies in the year after childbirth^7^ and fewer psychiatric hospitalizations in the 6 months after childbirth,^21^ which may be due to confounding by a higher severity of psychiatric conditions in individuals continuing use. Conversely, another study found that individuals who discontinued antidepressants had an increased risk of psychiatric emergencies in the 6 months after childbrith.^8^

Reasons for discontinuing SSRIs or SNRIs during pregnancy can include wishing to avoid fetal exposure, resolution of symptoms of depression or anxiety, or worsening of symptoms, resulting in a possible switch to other psychotropic medications. However, information on the reason for discontinuation is not available in health registers, and so we could not account for it in this study. Additionally, SSRI or SNRI discontinuation can occur at different times during pregnancy, but estimating the precise timing of discontinuation using data from prescription fills is not possible. The different reasons for and variable timing of SSRI or SNRI discontinuation result in heterogeneity in the discontinued use group, which may have differentially affected our findings. For example, women who discontinue despite having symptoms of depression or anxiety will be at risk of their symptoms worsening during pregnancy and could ultimately increase the number of postpartum psychiatric events observed in the discontinued use group. Furthermore, worsening depression or anxiety after discontinuation may result in reinitiation of SSRIs or SNRIs before the end of pregnancy. Notably, women who reinitiated treatment were likely classified in the SSRI or SNRI continued use group in this study.

Moreover, women who discontinue SSRIs or SNRIs during pregnancy likely have less severe symptoms of depression and anxiety and continue to do so after discontinuation. This may be one explanation for the lower HRs for outpatient visits, which could be interpreted as a decreased need in the discontinued use group and an increased need in the continued use group for planned follow-up psychiatric care in the postpartum period. This scenario illustrates that despite restricting our study to a cohort of women without severe or comorbid conditions, a difference in the severity of depression and anxiety in the SSRI or SNRI continued and discontinued use group may have still influenced our results. Given that the register data do not contain detailed information about disease severity, residual confounding owing to a higher severity of depression and anxiety in the continued use group could have negatively biased associations between SSRI or SNRI discontinuation and adverse postpartum psychiatric events.

Our findings showed no evidence overall of an association between SSRI or SNRI discontinuation and adverse postpartum psychiatric events. However, some estimates were high with large CIs (eg, for hospitalization due to psychosis at 90 days after childbirth, the aHR was 1.74; 95% CI, 0.72-4.22) but based on a small numbers of events.

Regarding sick leave absence, an internet-based study of more than 6000 women in 12 European countries found that two-thirds of participants with mood disorders reported being on sick leave during pregnancy and were more likely to have done so if they were not taking medication.^22^ To our knowledge, our study is the first population-based study to examine sick leave absence in pregnant women with prepregnancy SSRI or SNRI use. In our study cohort, 30.4% of women were on sick leave absence in the year prior to the start of pregnancy, while 47.6% were on sick leave absence during pregnancy. The proportion of women on sick leave in the postpartum period was similar between SSRI or SNRI continued and discontinued use groups, as was the proportion of individuals with a psychiatric indication for sick leave. However, individuals who continued SSRIs or SNRIs used more sick leave days, and a larger proportion used more than 90 days of sick leave, which may reflect a higher psychiatric burden.

### Limitations

This study has several limitations. First, we used an unsupervised clustering method to group SSRI and SNRI use patterns during pregnancy, and our clinical interpretation was that the trajectories group with the lower mean number of days covered by medication in pregnancy reflected women discontinuing SSRIs or SNRIs. There remains within-group heterogeneity and uncertainty related to whether women were truly discontinuing, lowering daily medication intake, interrupting, or continuing their medication.^17,18^ Misclassification of some individuals who continued SSRIs or SNRIs as discontinuing would result in higher prevalence of, for example, outcomes related to psychiatric outpatient care use in the discontinued use group, thereby attenuating estimates toward the null. However, evidence toward accurate clustering includes that more than half of women (53.5%) grouped as discontinuing did not have a prescription fill for an SSRI or SNRI after childbirth and among those that did, the median time to the first SSRI or SNRI prescription fill after childbirth was 5.4 months (162 days). Furthermore, an advantage of using an unsupervised clustering method was that it enabled a visual examination of clinically relevant SSRI or SNRI use trajectories over pregnancy, which is not possible using a fixed time window of SSRI or SNRI use.^23^ Second, we likely did not exclude all women with comorbid psychiatric conditions owing to having only 1 year of look-back prior to the start of pregnancy and because Swedish national health registers do not contain data from primary care or psychological counseling. However, we identified and excluded individuals with mental illness requiring recent specialist care, which may be associated with a large increase in risk for the measured outcomes. Similarly, we likely did not capture all psychiatric-related outcomes but did capture the most severe such outcomes. If women who discontinue SSRI or SNRI use in pregnancy have less severe symptoms of depression and anxiety, follow-up care is more likely to be in a primary care setting in the postpartum period and thus not captured. This may further explain the higher prevalence of psychiatric-related outpatient visits in the SSRI or SNRI continued use group relative to discontinued use group. However, we captured psychiatric outcomes requiring specialized care, which have the highest burden on women and their infants, and we found no evidence for a change in guidelines for SSRI or SNRI treatment during pregnancy.

## Conclusions

Reassuringly, this cohort study did not find evidence of an association between discontinuation of SSRIs or SNRIs and adverse psychiatric-related outcomes after childbirth among pregnant women with depression or anxiety and no recorded comorbid or severe psychiatric conditions. Outcomes included psychiatric-related hospitalizations or outpatient visits, suicidal behavior, or sick leave absence up to 1.5 years after childbirth.

## Notes

Supplement 1. **eTable 1.** Study population inclusion and exclusion criteria **eTable 2.** Outcome definitions **eFigure.** Clustering of trajectories of SSRI/SNRI use
eReferences.

Supplement 2.
Data Sharing Statement
