# Supplementary material for: Postpartum Psychiatric Outcomes and Sick Leave After Discontinuing SSRI or SNRI in Pregnancy
Source: JAMA Netw Open. 2024 Oct 8;7(10):e2438269. doi: 10.1001/jamanetworkopen.2024.38269 (PMC11581648; doi:10.1001/jamanetworkopen.2024.38269)
Supplement: Supplement 1. — eTable 1. Study population inclusion and exclusion criteria eTable 2. Outcome definitions eFigure. Clustering of trajectories of SSRI/SNRI use eReferences. [file jamanetwopen-e2438269-s001.pdf]

Supplementary Material

Cesta CE, Cohen JM, Eriksson J, Furu K, Zoega H, Pazzagli L. Postpartum psychiatric outcomes and sick leave after discontinuing SSRI or SNRI in pregnancy. *JAMA Netw Open*. 2024;7(10):e2438269. doi:10.1001/jamanetworkopen.2024.38269

eTable 1. Study population inclusion and exclusion criteria
eTable 2. Outcome definitions
eFigure. Clustering of trajectories of SSRI/SNRI use
eReferences.

eTable 1: Study population inclusion and exclusion criteria

	**Criteria**	**Details**
**Inclusion**	Singleton pregnancies	Remove multi-fetal pregnancies
**Inclusion**	Valid gestational age	Gestational age between 22-44 weeks and not missing
**Inclusion**	Residing in the country for the entire follow-up period	1 year prior to LMP to 1.5 years after childbirth
**Inclusion**	≥1 prescription fill of an SSRI or SNRI in the 90 days prior to LMP	**ATC codes:**<br>*N06AB* SSRI<br>*N06AX21* SNRI, duloxetine<br>*N06AX16* SNRI, venlafaxine<br>*note the SNRIs *N06AX23* (desvenlafaxine) and *N06AX17* (milnacipran) are not approved for use in Sweden.
**Exclusion**	≥1 psychiatric diagnosis in the year before LMP, **except** outpatient diagnoses of unipolar depression and anxiety	Diagnoses within ICD-10 Chapter F<br>**except** outpatient diagnoses of:<br>*F32.0* Mild depressive episode<br>*F32.1* Moderate depressive episode<br>*F32.8* Other depressive episodes<br>*F32.9* Depressive episode, unspecified<br>(hence, pregnancies with outpatient diagnoses of *F32.2/F32.3 Severe depressive episode without/with psychotic symptoms* were excluded)<br>*F33* Recurrent depressive disorder<br>*F34* Persistent mood [affective] disorders<br>*F40-48* Neurotic, stress-related and somatoform disorders
**Exclusion**	≥1 prescription fill of non-SSRI/SNRI antidepressants, antipsychotics including lithium, or antiepileptics	*N06AF* Monoamine oxidase inhibitors, non-selective<br>*N06AG* Monoamine oxidase inhibitors, non-selective<br>*N06AX* Other (except SNRI: N06AX21, N06AX16)<br>*N05A* Antipsychotics<br>*N03* Antiepileptics
**Exclusion (for sick leave outcome only)**	Women on long-term disability	Not eligible for sick-leave benefits

Abbreviation: LMP, first day of the last menstrual period; SSRI, Selective serotonin reuptake inhibitors; SNRI, Serotonin–norepinephrine reuptake inhibitors.

© 2024 Cesta CE et al. *JAMA Network Open*.

eTable 2: Outcome definitions

Outcome	Description
**Any psychiatric diagnosis**	Main diagnosis: ICD-10 F10-F99
**Psychosis (1)**	Main diagnosis: ICD-10 F20-F31, F32.3, F33.3, F53.1
**Depression or anxiety (2)**	Main diagnosis: ICD-10 F32-F39, F53.0, F40-F48
**Self-harm (3)**	Main or secondary diagnosis:<br>ICD-10 X60-X84, Y10-Y34
**Death, any cause**	Identified by a record of death in the Cause of Death Register with any ICD-10 code for cause of death
**Death, suicide**	Identified by a record of death in the Cause of Death Register with the following ICD-10 codes for cause of death:<br>X60-X84 and Y10-Y34
**Sick leave absence**	A sick leave episode with a starting date after the date of childbirth
**Psychiatric indication for sick leave absence**	ICD-10 code associated with sick leave episode:<br>F10-F99, X60-X84
**Number of days of sick leave absence**	Number of days covered by sick leave (multiple episodes allowed) between date of childbirth+1 day to date of childbirth+1.5 years
**Restarting SSRI/SNRI after childbirth (descriptive outcome for discontinuers)**	At least 1 prescription fill of an SSRI/SNRI after the date of childbirth and date of childbirth+1.5 years.

Abbreviation: SSRI, Selective serotonin reuptake inhibitors; SNRI, Serotonin–norepinephrine reuptake inhibitors.

© 2024 Cesta CE et al. *JAMA Network Open*.

eFigure 1: Clustering of trajectories of SSRI/SNRI use

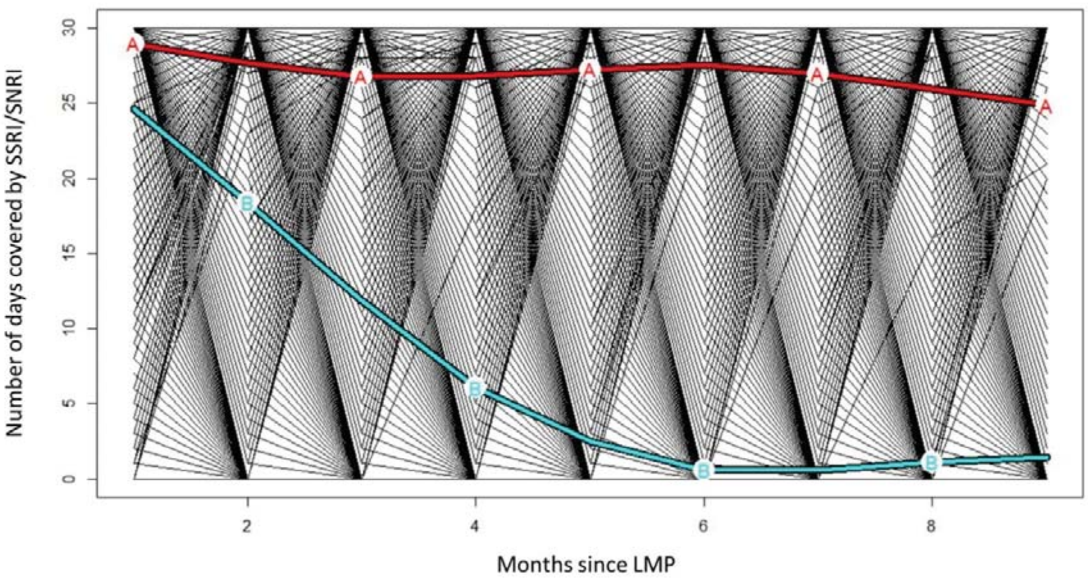

© 2024 Cesta CE et al. *JAMA Network Open*.

References

1. Warselius P, Cnattingius S, Li J, Wei D, Valdimarsdottir UA, Kosidou K, et al. Maternal bereavement shortly before or during pregnancy and risk of postpartum psychotic illness: a population-based study from Denmark and Sweden. Clin Epidemiol. 2019;11:285-98.
2. Liu C, Butwick A, Sand A, Wikström AK, Snowden JM, Stephansson O. The association between postpartum hemorrhage and postpartum depression: A Swedish national register-based study. PLoS One. 2021;16(8):e0255938.
3. Reutfors J, Andersson TM, Brenner P, Brandt L, DiBernardo A, Li G, et al. Mortality in treatment-resistant unipolar depression: A register-based cohort study in Sweden. J Affect Disord. 2018;238:674-9.

© 2024 Cesta CE et al. *JAMA Network Open*.
