# Supplementary material for: Postpartum Psychiatric Outcomes and Sick Leave After Discontinuing SSRI or SNRI in Pregnancy
Source: JAMA Netw Open. 2024 Oct 8;7(10):e2438269. doi: 10.1001/jamanetworkopen.2024.38269 (PMC11581648; doi:10.1001/jamanetworkopen.2024.38269)
Supplement: Supplement 2. — Data Sharing Statement [file jamanetwopen-e2438269-s002.pdf]

Data Sharing Statement

Cesta. Postpartum Psychiatric Outcomes and Sick Leave After Discontinuing SSRI or SNRI in Pregnancy. *JAMA Netw Open*. Published online October 8, 2024. doi:10.1001/jamanetworkopen.2024.38269

Data
Data available: No

Additional Information
Explanation for why data not available: Due to data privacy laws, the data cannot be made publicly available
